# Supplementary material for: Pharmaceutical marketing and patient trust: How do doctor-targeted campaigns affect patients?
Source: PLoS One. 2026 Mar 27;21(3):e0344509. doi: 10.1371/journal.pone.0344509 (PMC13028511; doi:10.1371/journal.pone.0344509)
Supplement: Attachment — Submitted filename: Response to Reviewer 2_last.docx [file pone.0344509.s002.docx]

Dear Reviewer 2,

Thank you very much for the very helpful review of our article ‘Pharmaceutical Marketing and Patient Trust: How Do Doctor-Targeted Campaigns Affect Patients?’. We have sought to address your important points as comprehensively as possible. Our responses to your specific comments are provided below.

Remark #1

*The information on the impact of each factor on causing it to be noticed is interesting, and they effects on how the patients view each type of promotional action is well done.*

Thank you very much for appreciating our work.

Remark #2

*I would suggest limiting your discussion on the low r2 of the regression and admit it wasn't a great model, instead of the in-depth analysis. Does your background theory suggest any other possible model for which you have measures? It the dependent variable really the one that you want to know about? Think about another regression equation or minimize the discussion of that measure.*

Following a series of additional analyses – none of which yielded satisfactory improvements – we retained the weak regression model but substantially shortened its description. We also removed the regression-focused section of the Discussion. Instead, we emphasize a key takeaway: sociodemographic variables alone are weak predictors of patients’ attention to pharmaceutical marketing; a fuller account likely requires richer psychological and situational variables. We believe these revisions improve clarity and better align the manuscript with the evidence. The text is now as follows:

Discussion (only this was left)

‘Our regression model linking demographic factors to the detection of pharmaceutical marketing exhibited low explanatory power, and the observed associations had small effect sizes. This suggests that socio-demographic characteristics alone have limited predictive value in this dataset and specification. More promising predictors may include: attitudes toward the industry, health literacy, health anxiety, locus of control, exposure to information sources, and the patient–physician relationship, which were not included here. These considerations point to important directions for future research.’

Future Research

‘Subsequent analyses should also incorporate richer psychological and relational constructs (e.g., attitudes toward the industry, health literacy, health anxiety, locus of control, exposure to information sources, and the patient–physician relationship). By contrast, conventional sociodemographic variables, which explained little in our data, appear to offer limited explanatory value on their own.’

We hope that these changes are acceptable. If you have any further comments we would be happy to address them.

Sincerely,

Authors
